# Supplementary material for: Pharmacogenetics of Osteoporosis: A Pathway Analysis of the Genetic Influence on the Effects of Antiresorptive Drugs
Source: Pharmaceutics. 2022 Apr 2;14(4):776. doi: 10.3390/pharmaceutics14040776 (PMC9032991; doi:10.3390/pharmaceutics14040776)
Supplement: Supplementary file 1 [file pharmaceutics-14-00776-s001.zip › pharmaceutics-1647011-supplementary.pdf]

pharmaceutics

MDPI

Supplementary Materials: Pharmacogenetics of Osteoporosis: A Pathway Analysis of the Genetic Influence on the Effects of Antiresorptive Drugs

Álvaro del Real, Carmen Valero, Jose M. Olmos, José L. Hernández and José A. Riancho

Table S1. Genes and number of SNPs studied.

Gene	Pathway	Number of SNPs
ARSD	Oestrogen	20
ARSL	Estrogen	9
CYP19A1	Estrogen	42
CYP1A1	Estrogen	4
CYP1A2	Estrogen	16
CYP1B1	Estrogen	56
CYP2D6	Estrogen	14
CYP3A4	Estrogen	14
EGF	Estrogen	26
ELK1	Estrogen	1
ERK1	Estrogen	15
ESR1	Estrogen	91
ESR2	Estrogen	13
FOS	Estrogen	39
GPER1	Estrogen	9
HSD17B1	Estrogen	2
IKBKB	Estrogen	6
IKBKG	Estrogen	1
JUN	Estrogen	15
MAP2K1	Estrogen	10
MAPK1	Estrogen	15
MAPK14	Estrogen	9
MAPK8	Estrogen	20
MAPK9	Estrogen	22
NFKB1	Estrogen	35
SP1	Estrogen	9
STS	Estrogen	41
SULT1A1	Estrogen	4
SULT1E1	Estrogen	21
SULT2A1	Estrogen	19
UGT1A1	Estrogen	7
UGT1A8	Estrogen	22
UGT1A9	Estrogen	38
UGT2B7	Estrogen	29
ACAT1	Mevalonate	3
ACAT2	Mevalonate	7
DHDDS	Mevalonate	3
FDPS	Mevalonate	4

FNTA	Mevalonate	4
FNTB	Mevalonate	31
HMGCR	Mevalonate	9
HMGCS1	Mevalonate	4
HMGCS2	Mevalonate	5
ICMT	Mevalonate	2
IDI1	Mevalonate	3
IDI2	Mevalonate	5
MVD	Mevalonate	8
MVK	Mevalonate	6
NUS1	Mevalonate	4
PCYOX1	Mevalonate	1
PDSS1	Mevalonate	21
PDSS2	Mevalonate	27
PMVK	Mevalonate	2
RCE1	Mevalonate	1
ZMPSTE24	Mevalonate	4
